# Bis(guanidinium) tetra­iodidomercurate(II)

**DOI:** 10.1107/S160053680901280X

**Published:** 2009-04-10

**Authors:** Hiromitsu Terao, Thorsten M. Gesing, Hideta Ishihara, Yoshihiro Furukawa, B. Thimme Gowda

**Affiliations:** a Faculty of Integrated Arts and Sciences, Tokushima University, Minamijosanjima-cho, Tokushima 770-8502, Japan; b FB05 Kristallographie, Universität Bremen, Klagenfurther Strasse, 28359 Bremen, Germany; c Faculty of Culture and Education, Saga University, Saga 840-8502, Japan; d Graduate School of Education, Hiroshima University, Higashi-Hiroshima 739-8524, Japan; e Department of Chemistry, Mangalore University, Mangalagangotri 574 199, Mangalore, India

## Abstract

The Hg atom in the crystal structure of the title compound, (CH_6_N_3_)_2_[HgI_4_], is tetra­hedrally coordinated by four I atoms. The [HgI_4_]^2−^ ions are inter­connected to the [C(NH_2_)_3_]^+^ ions by N—H⋯I hydrogen bonds, forming a three-dimensional network. The four different observed Hg—I distances [2.760 (2), 2.7762 (15), 2.8098 (14) and 2.833 (2) Å] are consistent with four different ^127^I NQR frequencies observed, showing the existence of four unique I atoms in the tetra­iodidomercurate unit.

## Related literature

For synthetic methods, see: Furukawa *et al.* (2005 ▶); For the ability of the guanidinium ion to make hydrogen bonds and its unique planar shape, see: Terao *et al.* (2000 ▶). Hg–halogen bonds are sensitive to inter­molecular inter­actions such as hydrogen bonding (Ishihara *et al.*, 2002 ▶), as evidenced by the halogen NQR of Hg compounds in which the resonance frequencies are widely spread (Furukawa *et al.*, 2005 ▶). For background to this study, see: Terao *et al.* (2009 ▶).
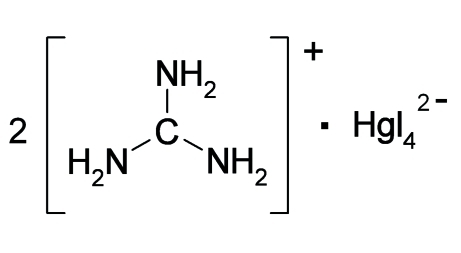


## Experimental

### 

#### Crystal data

(CH_6_N_3_)_2_[HgI_4_]
*M* *_r_* = 828.37
Triclinic, 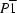

*a* = 8.981 (2) Å
*b* = 8.996 (2) Å
*c* = 12.302 (3) Å
α = 105.80 (3)°
β = 95.79 (4)°
γ = 118.46 (2)°
*V* = 808.9 (5) Å^3^
*Z* = 2
Mo *K*α radiation
μ = 17.13 mm^−1^
*T* = 298 K
0.42 × 0.38 × 0.32 mm

#### Data collection

Stoe IPDS-I diffractometer
Absorption correction: numerical (*X-SHAPE*; Stoe & Cie, 1999 ▶) *T*_min_ = 0.017, *T*_max_ = 0.057
14500 measured reflections
3613 independent reflections
1846 reflections with *I* > 2σ(*I*)
*R*_int_ = 0.118

#### Refinement

*R*[*F* ^2^ > 2σ(*F* ^2^)] = 0.059
*wR*(*F* ^2^) = 0.135
*S* = 0.81
3613 reflections
156 parameters
32 restraints
H atoms treated by a mixture of independent and constrained refinement
Δρ_max_ = 3.08 e Å^−3^
Δρ_min_ = −2.71 e Å^−3^

### 

Data collection: *EXPOSE* (Stoe & Cie, 1999 ▶); cell refinement: *CELL* (Stoe & Cie, 1999 ▶); data reduction: *XPREP* (Bruker, 2003 ▶); program(s) used to solve structure: *SHELXS86* (Sheldrick, 2008 ▶); program(s) used to refine structure: *SHELXL93* (Sheldrick, 2008 ▶); molecular graphics: *DIAMOND* (Crystal Impact, 2008 ▶) and *PLATON* (Spek, 2009 ▶); software used to prepare material for publication: *SHELXL93*.

## Supplementary Material

Crystal structure: contains datablocks I, global. DOI: 10.1107/S160053680901280X/bx2201sup1.cif

Structure factors: contains datablocks I. DOI: 10.1107/S160053680901280X/bx2201Isup2.hkl

Additional supplementary materials:  crystallographic information; 3D view; checkCIF report

## Figures and Tables

**Table 1:** Hydrogen-bond geometry (Å, °)

*D*—H⋯*A*	*D*—H	H⋯*A*	*D*⋯*A*	*D*—H⋯*A*
N11—H11*A*⋯I1^i^	0.87 (4)	3.00 (4)	3.78 (2)	151 (2)
N12—H12*A*⋯I2	0.87 (4)	3.46 (2)	3.83 (2)	123 (2)
N13—H13*A*⋯I3^ii^	0.87 (4)	2.96 (4)	3.80 (2)	161 (2)
N13—H13*B*⋯I1^i^	0.87 (4)	2.88 (4)	3.69 (2)	156 (2)
N21—H21*A*⋯I3^iii^	0.87 (4)	3.03 (4)	3.82 (2)	151 (2)
N21—H21*B*⋯I2	0.87 (4)	2.91 (4)	3.74 (2)	162 (6)
N22—H22*A*⋯I4^iv^	0.87 (9)	2.98 (4)	3.82 (2)	162 (2)
N22—H22*B*⋯I3^iii^	0.87 (10)	3.05 (4)	3.81 (2)	147 (2)
N23—H23*A*⋯I4^iv^	0.87 (9)	2.91 (4)	3.71 (2)	153 (2)
N23—H23*B*⋯I2	0.87 (4)	2.99 (4)	3.82 (2)	161 (6)

Symmetry codes: (i) 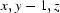; (ii) 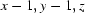; (iii) 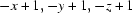; (iv) 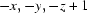.

**Fig. 1.**
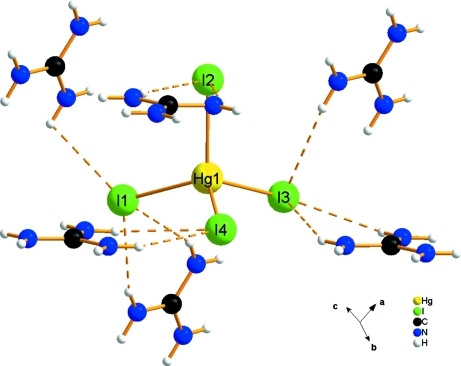
Molecular structure of (I), showing the atom labeling scheme. The displacement ellipsoids are drawn at the 50% probability level. The H atoms are represented as small spheres of arbitrary radii.

**Fig. 2.**
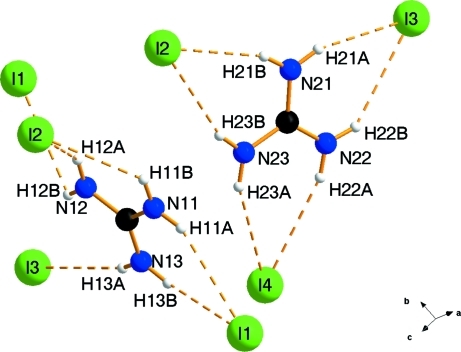
Two distinct guanidinium ions in the crystal structure of (I) along with the numbering of the atoms.

**Fig. 3.**
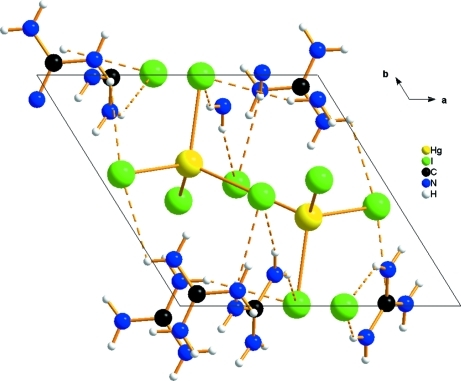
Packing diagram of (I) as viewed in the direction of c axis.

## supplementary crystallographic information

### Comment

The ability of guanidium ion, [C(NH_2_)_3_]^+^ in making hydrogen bonds and its unique planar shape has been recognized (Terao *et al.*, 2000). Further, the guanidium ions tend to undergo reorientation motions about their (pseudo) C_3_ axes in the crystals. Due to the soft nature, Hg atoms are amenable to polarization and thus the Hg-halogen bonds are sensitive to the intermolecular interactions such as hydrogen bonding (Ishihara *et al.*, 2002). This was evident in the halogen NQR of the Hg compounds in which the resonance frequencies are widely spread (Furukawa *et al.*, 2005). Thus the study of the structure and bonding of this class of compounds is interesting. As a part of our investigations in this direction (Terao *et al.*, 2009), we report herein the crystal structure of Guanidinium tetraiodomercurate(II) (I). In the structure, the mercury atom is tetrahedrally coordinated by four iodine atoms and the resulting HgI_4_ tetrahedra are interconnected to the [C(NH_2_)_3_]^+^ ions by iodine-hydrogen bonds forming a three-dimensional network (Fig. 1). Four different Hg—I distances were observed which are consistent with four different I-127 NQR frequencies observed (Furukawa *et al.*, 2005), establishing the existence of four inequivalent I atoms in the tetraiodomercurate unit. The packing diagram of the crystal structure, as viewed in the direction of *c* axis is shown in Fig. 3.

### Experimental

Guanidinium tetraiodomercurate(II) was prepared by slow concentration of methanolic solution containing mercuric iodide (0.01 mol, 4.54 g) and guanidium iodide (0.024 mol, 4.48 g) in slightly more than 1:2 molar ratio. The purity of the compound was checked by elemental analysis and characterized by its NMR and NQR spectra (Furukawa *et al.*, 2005). The single crystals used in X-ray diffraction studies were grown in methanolic solution by a slow evaporation at room temperature.

### Refinement

The N—H distances were restrained to 0.87–0.88 Å and the coordinates of the H atoms were refined with isotropic displacement parameters set to 1.2 times of the *U*_eq_ of the parent atom.

### Crystal data

**Table:**

(CH_6_N_3_)_2_[HgI_4_]	*Z* = 2
*M**_r_* = 828.37	*F*(000) = 716
Triclinic, *P*1	*D*_x_ = 3.401 Mg m^−^^3^
Hall symbol: -P 1	Mo *K*α radiation, λ = 0.71073 Å
*a* = 8.981 (2) Å	Cell parameters from 2000 reflections
*b* = 8.996 (2) Å	θ = 2.7–28.0°
*c* = 12.302 (3) Å	µ = 17.13 mm^−^^1^
α = 105.80 (3)°	*T* = 298 K
β = 95.79 (4)°	Cylindric, yellow
γ = 118.46 (2)°	0.42 × 0.38 × 0.32 mm
*V* = 808.9 (5) Å^3^	

### Data collection

**Table:**

Stoe IPDS-I diffractometer	3613 independent reflections
Radiation source: fine-focus sealed tube	1846 reflections with *I* > 2σ(*I*)
graphite	*R*_int_ = 0.118
imaging plate dynamic profile intergration scans	θ_max_ = 28.0°, θ_min_ = 2.7°
Absorption correction: numerical (*X-SHAPE*; Stoe & Cie, 1999)	*h* = −11→11
*T*_min_ = 0.017, *T*_max_ = 0.057	*k* = −11→11
14500 measured reflections	*l* = −16→16

### Refinement

**Table:**

Refinement on *F*^2^	Secondary atom site location: difference Fourier map
Least-squares matrix: full	Hydrogen site location: inferred from neighbouring sites
*R*[*F*^2^ > 2σ(*F*^2^)] = 0.059	H atoms treated by a mixture of independent and constrained refinement
*wR*(*F*^2^) = 0.135	*w* = 1/[σ^2^(*F*_o_^2^) + (0.0353*P*)^2^] where *P* = (*F*_o_^2^ + 2*F*_c_^2^)/3
*S* = 0.81	(Δ/σ)_max_ < 0.001
3613 reflections	Δρ_max_ = 3.08 e Å^−^^3^
156 parameters	Δρ_min_ = −2.71 e Å^−^^3^
32 restraints	Extinction correction: *SHELXL93* (Sheldrick, 2008), Fc^*^=kFc[1+0.001xFc^2^λ^3^/sin(2θ)]^-1/4^
Primary atom site location: structure-invariant direct methods	Extinction coefficient: 0.00075 (10)

### Special details

**Table:**

Geometry. All e.s.d.'s (except the e.s.d. in the dihedral angle between two l.s. planes) are estimated using the full covariance matrix. The cell e.s.d.'s are taken into account individually in the estimation of e.s.d.'s in distances, angles and torsion angles; correlations between e.s.d.'s in cell parameters are only used when they are defined by crystal symmetry. An approximate (isotropic) treatment of cell e.s.d.'s is used for estimating e.s.d.'s involving l.s. planes.
Refinement. Refinement of *F*^2^ against ALL reflections. The weighted *R*-factor *wR* and goodness of fit *S* are based on *F*^2^, conventional *R*-factors *R* are based on *F*, with *F* set to zero for negative *F*^2^. The threshold expression of *F*^2^ > σ(*F*^2^) is used only for calculating *R*-factors(gt) *etc*. and is not relevant to the choice of reflections for refinement. *R*-factors based on *F*^2^ are statistically about twice as large as those based on *F*, and *R*- factors based on ALL data will be even larger.

### Fractional atomic coordinates and isotropic or equivalent isotropic displacement parameters (Å^2^)

**Table:**

	*x*	*y*	*z*	*U*_iso_*/*U*_eq_	
Hg1	0.34641 (9)	0.61562 (10)	0.73508 (6)	0.0667 (3)	
I1	0.0809 (2)	0.5696 (2)	0.84518 (10)	0.0650 (3)	
I2	0.53156 (14)	0.4740 (2)	0.82071 (9)	0.0607 (3)	
I3	0.58185 (14)	0.9944 (2)	0.80058 (10)	0.0627 (3)	
I4	0.22651 (14)	0.4550 (2)	0.49342 (9)	0.0673 (3)	
C1	0.0864 (17)	0.0540 (15)	0.8824 (8)	0.051 (3)	
N11	0.2387 (17)	0.0661 (18)	0.9149 (13)	0.072 (4)	
H11A	0.246 (12)	−0.030 (7)	0.902 (4)	0.12 (3)*	
H11B	0.333 (7)	0.174 (5)	0.9499 (19)	0.12 (3)*	
N12	0.0824 (14)	0.2000 (14)	0.9034 (11)	0.066 (4)	
H12A	0.179 (2)	0.3056 (13)	0.9387 (16)	0.12 (3)*	
H12B	−0.0169 (19)	0.193 (2)	0.8825 (17)	0.12 (3)*	
N13	−0.0542 (16)	−0.1065 (19)	0.8300 (14)	0.080 (4)	
H13A	−0.152 (4)	−0.111 (8)	0.810 (3)	0.12 (3)*	
H13B	−0.054 (9)	−0.207 (5)	0.815 (3)	0.12 (3)*	
C2	0.2590 (18)	−0.012 (2)	0.5152 (16)	0.067 (4)	
N21	0.4067 (18)	0.136 (2)	0.5198 (14)	0.086 (5)	
H21A	0.453 (8)	0.138 (11)	0.461 (4)	0.12 (3)*	
H21B	0.457 (8)	0.232 (6)	0.584 (3)	0.12 (3)*	
N22	0.1800 (17)	−0.158 (2)	0.4211 (13)	0.092 (5)	
H22A	0.085 (3)	−0.246 (11)	0.427 (11)	0.12 (3)*	
H22B	0.212 (14)	−0.173 (18)	0.357 (5)	0.12 (3)*	
N23	0.193 (2)	−0.009 (3)	0.6078 (14)	0.110 (7)	
H23A	0.098 (3)	−0.100 (11)	0.610 (12)	0.12 (3)*	
H23B	0.252 (13)	0.093 (8)	0.668 (7)	0.12 (3)*	

### Atomic displacement parameters (Å^2^)

**Table:**

	*U*^11^	*U*^22^	*U*^33^	*U*^12^	*U*^13^	*U*^23^
Hg1	0.0686 (4)	0.0651 (5)	0.0641 (4)	0.0319 (4)	0.0196 (3)	0.0262 (4)
I1	0.0802 (7)	0.0566 (7)	0.0743 (7)	0.0413 (6)	0.0367 (6)	0.0307 (6)
I2	0.0632 (6)	0.0535 (7)	0.0608 (6)	0.0271 (6)	0.0115 (5)	0.0233 (5)
I3	0.0605 (6)	0.0547 (7)	0.0725 (7)	0.0284 (6)	0.0193 (5)	0.0259 (6)
I4	0.0606 (6)	0.0668 (8)	0.0540 (6)	0.0182 (6)	0.0137 (5)	0.0237 (6)
C1	0.060 (9)	0.039 (10)	0.050 (8)	0.023 (8)	0.014 (7)	0.017 (8)
N11	0.063 (9)	0.069 (11)	0.111 (12)	0.045 (8)	0.032 (9)	0.047 (10)
N12	0.063 (8)	0.039 (9)	0.077 (9)	0.022 (7)	−0.004 (7)	0.009 (8)
N13	0.068 (9)	0.053 (11)	0.113 (13)	0.033 (9)	0.004 (9)	0.027 (10)
C2	0.053 (9)	0.047 (12)	0.076 (12)	0.014 (9)	0.003 (9)	0.016 (10)
N21	0.079 (10)	0.048 (11)	0.088 (11)	0.005 (9)	0.033 (9)	0.013 (9)
N22	0.076 (10)	0.054 (12)	0.060 (9)	−0.012 (9)	0.006 (8)	−0.006 (8)
N23	0.074 (11)	0.096 (15)	0.077 (11)	−0.007 (10)	0.032 (10)	0.010 (11)

### Geometric parameters (Å, °)

**Table:**

Hg1—I4	2.760 (2)	N11—H11A	0.88 (8)
Hg1—I1	2.7762 (15)	N11—H11B	0.87 (5)
Hg1—I2	2.8098 (14)	N12—H12A	0.87 (2)
Hg1—I3	2.833 (2)	N12—H12B	0.87 (2)
I1—H13B^i^	2.87 (7)	N13—H13A	0.87 (5)
I1—H11A^i^	3.00 (4)	N13—H13B	0.88 (6)
I2—H21B	2.91 (3)	C2—N22	1.30 (2)
I2—H23B	2.99 (5)	C2—N21	1.34 (2)
I3—H13A^ii^	2.97 (5)	C2—N23	1.34 (2)
I3—H22B^iii^	3.05 (7)	N21—H21B	0.87 (4)
I3—H21A^iii^	3.03 (4)	N21—H21A	0.87 (7)
I3—H12B^ii^	3.057 (19)	N22—H22B	0.87 (9)
C1—N13	1.29 (2)	N22—H22A	0.87 (9)
C1—N12	1.29 (2)	N23—H23A	0.87 (9)
C1—N11	1.32 (2)	N23—H23B	0.87 (8)
			
I4—Hg1—I1	113.75 (5)	H13A—N13—H13B	120 (6)
I4—Hg1—I2	109.54 (5)	H13A—N13—C1	117.4 (42)
I1—Hg1—I2	108.81 (4)	H13B—N13—C1	122.5 (42)
I4—Hg1—I3	109.38 (6)	N22—C2—N21	120.3 (17)
I1—Hg1—I3	107.26 (5)	N22—C2—N23	119.8 (15)
I2—Hg1—I3	107.93 (5)	N21—C2—N23	119.9 (17)
N13—C1—N12	121.1 (14)	H21B—N21—H21A	120 (7)
N13—C1—N11	119.7 (14)	H21B—N21—C2	118.6 (57)
N12—C1—N11	119.2 (14)	H21A—N21—C2	121.3 (58)
H11A—N11—H11B	120 (7)	H22B—N22—H22A	120 (11)
H11A—N11—C1	120 (11)	H22B—N22—C2	126.5 (91)
H11B—N11—C1	118.4 (62)	H22A—N22—C2	113.5 (90)
H12A—N12—H12B	120 (2)	H23A—N23—H23B	120 (11)
H12A—N12—C1	119.9 (19)	H23A—N23—C2	125.3 (100)
H12B—N12—C1	120.0 (18)	H23B—N23—C2	114.6 (100)
			
N13—C1—N11—H11A	0.0 (6)	N22—C2—N21—H21B	180.0 (5)
N12—C1—N11—H11A	−180.0 (5)	N23—C2—N21—H21B	−0.1 (6)
N13—C1—N11—H11B	180.0 (6)	N22—C2—N21—H21A	0.0 (5)
N12—C1—N11—H11B	0.0 (5)	N23—C2—N21—H21A	179.9 (6)
N13—C1—N12—H12A	−180.0 (6)	N21—C2—N22—H22B	0.1 (6)
N11—C1—N12—H12A	0.0 (4)	N23—C2—N22—H22B	−179.9 (6)
N13—C1—N12—H12B	0.1 (9)	N21—C2—N22—H22A	180.0 (5)
N11—C1—N12—H12B	−180.0 (8)	N23—C2—N22—H22A	0.1 (6)
N12—C1—N13—H13A	−0.1 (10)	N22—C2—N23—H23A	−0.2 (11)
N11—C1—N13—H13A	179.9 (7)	N21—C2—N23—H23A	179.9 (8)
N12—C1—N13—H13B	−179.9 (7)	N22—C2—N23—H23B	−179.9 (7)
N11—C1—N13—H13B	0.1 (10)	N21—C2—N23—H23B	0.1 (9)

Symmetry codes: (i) *x*, *y*+1, *z*; (ii) *x*+1, *y*+1, *z*; (iii) −*x*+1, −*y*+1, −*z*+1.

### Hydrogen-bond geometry (Å, °)

**Table:**

*D*—H···*A*	*D*—H	H···*A*	*D*···*A*	*D*—H···*A*
N11—H11A···I1^iv^	0.87 (4)	3.00 (4)	3.78 (2)	151 (2)
N12—H12A···I2	0.87 (4)	3.46 (2)	3.83 (2)	123 (2)
N13—H13A···I3^v^	0.87 (4)	2.96 (4)	3.80 (2)	161 (2)
N13—H13B···I1^iv^	0.87 (4)	2.88 (4)	3.69 (2)	156 (2)
N21—H21A···I3^iii^	0.87 (4)	3.03 (4)	3.82 (2)	151 (2)
N21—H21B···I2	0.87 (4)	2.91 (4)	3.74 (2)	162 (6)
N22—H22A···I4^vi^	0.87 (9)	2.98 (4)	3.82 (2)	162 (2)
N22—H22B···I3^iii^	0.87 (10)	3.05 (4)	3.81 (2)	147 (2)
N23—H23A···I4^vi^	0.87 (9)	2.91 (4)	3.71 (2)	153 (2)
N23—H23B···I2	0.87 (4)	2.99 (4)	3.82 (2)	161 (6)

Symmetry codes: (iv) *x*, *y*−1, *z*; (v) *x*−1, *y*−1, *z*; (iii) −*x*+1, −*y*+1, −*z*+1; (vi) −*x*, −*y*, −*z*+1.

## References

[bb1] Bruker (2003). *XPREP* Bruker AXS Inc., Madison, Wisconsin, USA.

[bb2] Crystal Impact (2008). *DIAMOND* Crystal Impact GmbH, Bonn, Germany.

[bb3] Furukawa, Y., Terao, H., Ishihara, H., Gesing, T. M. & Buhl, J.-C. (2005). *Hyperfine Interact.* **159**, 143–148.

[bb4] Ishihara, H., Hatano, N., Horiuchi, K. & Terao, H. (2002). *Z. Naturforsch. Teil A*, **57**, 343–347.

[bb5] Sheldrick, G. M. (2008). *Acta Cryst.* A**64**, 112–122. 10.1107/S0108767307043930 18156677

[bb6] Spek, A. L. (2009). *Acta Cryst.* D**65**, 148–155. 10.1107/S090744490804362X PMC2631630 19171970

[bb7] Stoe & Cie. (1999). *EXPOSE*, *CELL* and *X-SHAPE* Stoe & Cie GmbH, Darmstadt, Germany.

[bb8] Terao, H., Gesing, T. M., Ishihara, H., Furukawa, Y. & Gowda, B. T. (2009). *Acta Cryst.* E**65**, m323. 10.1107/S1600536809005972 PMC2968626 21582096

[bb9] Terao, H., Hashimoto, M., Hashimoto, A. & Furukawa, Y. (2000). *Z. Naturforsch. Teil A*, **55**, 230–236.

